# Critical appraisal of the 2020 ESC guideline recommendations on diagnosis and risk assessment in patients with suspected non-ST-segment elevation acute coronary syndrome

**DOI:** 10.1007/s00392-021-01821-2

**Published:** 2021-02-26

**Authors:** Evangelos Giannitsis, Stefan Blankenberg, Robert H. Christenson, Norbert Frey, Stephan von Haehling, Christian W. Hamm, Kenji Inoue, Hugo A. Katus, Chien-Chang Lee, James McCord, Martin Möckel, Jack Tan Wei Chieh, Marco Tubaro, Kai C. Wollert, Kurt Huber

**Affiliations:** 1 Medizinische Klinik III, Department of Cardiology, Angiology and Pulmology, University Hospital of Heidelberg, Im Neuenheimer Feld 410, 69120 Heidelberg, Germany; 2 Department of Cardiology, University Heart and Vascular Centre Hamburg, Hamburg, Germany; 3 University of Maryland School of Medicine, Baltimore, MD USA; 4 Department of Cardiology and Pneumology, University of Göttingen Medical Center, Göttingen, Germany; 5 German Center for Cardiovascular Research (DZHK), Partner Site Göttingen, Göttingen, Germany; 6 Department of Cardiology, Kerckhoff Heart and Thorax Centre, Bad Nauheim, Germany; 7 Department of Cardiovascular Biology and Medicine, Juntendo University Nerima Hospital, Tokyo, Japan; 8 Department of Emergency Medicine, National Taiwan University Hospital, Taipei, Taiwan; 9 Henry Ford Heart and Vascular Institute Detroit, Detroit, MI USA; 10 Department of Emergency Medicine, Charité-Universitätsmedizin Berlin, Campus Mitte and Virchow, Berlin, Germany; 11 Department of Cardiology, National Heart Centre and Sengkang General Hospital, Singapore, Singapore; 12 ICCU, San Filippo Neri Hospital, Rome, Italy; 13 Division of Molecular and Translational Cardiology, Department of Cardiology and Angiology, Hannover Medical School, Hannover, Germany; 14 3rd Department of Internal Medicine, Cardiology and Intensive Care Medicine, Wilhelminenhospital, Vienna, Austria; 15 Medical School, Sigmund Freud University, Vienna, Austria

**Keywords:** Guidelines, Critical appraisal, Acute coronary syndromes, Non-ST-segment elevation, High-sensitivity troponin, Diagnosis, Prognosis, Management

## Abstract

Multiple new recommendations have been introduced in the 2020 ESC guidelines for the management of acute coronary syndromes with a focus on diagnosis, prognosis, and management of patients presenting without persistent ST-segment elevation. Most recommendations are supported by high-quality scientific evidence. The guidelines provide solutions to overcome obstacles presumed to complicate a convenient interpretation of troponin results such as age-, or sex-specific cutoffs, and to give practical advice to overcome delays of laboratory reporting. However, in some areas, scientific support is less well documented or even missing, and other areas are covered rather by expert opinion or subjective recommendations. We aim to provide a critical appraisal on several recommendations, mainly related to the diagnostic and prognostic assessment, highlighting the discrepancies between Guideline recommendations and the existing scientific evidence.

## Introduction

In August 2020, the European Society of Cardiology (ESC) presented the Guidelines on NSTE-ACS during the Annual Congress that was held on a virtual platform [1]. These 2020 Guidelines introduced new and revised sections on important topics that differ from the preceding 2015 ESC Guidelines on NSTE-ACS [2]. We believe that some of the new recommendations, which can be anticipated to influence medical decision-making, were not supported by an appropriate level of evidence and are worth reconsidering.

As a global phenomenon, the diagnosis of acute myocardial infarction (AMI) has remained challenging and still chest pain and/or dyspnea are amongst the most prevalent symptoms leading to emergency department (ED) admission in the USA [3]. Accordingly, many EDs face overcrowding as the numbers of patients seeking medical attention for unspecific chest pain are steadily increasing while numbers of patients with confirmed NSTE-ACS have remained stable or are slightly decreasing [4]. Acceleration of patient disposition and facilitation of safe and early discharge have been identified as pragmatic solutions to decongest busy EDs [5]. Therefore, the rationale to advance the use of hs-cTn assays and to further instigate the implementation of faster diagnostic algorithms has to be appreciated. Moreover, guidelines endorse measures for a more convenient, user-friendly interpretation of cTn results such as the recommendation to abstain from age-, sex-, or comorbidity-adapted decision cutoffs although the use of sex-specific cutoffs has been endorsed by the 4th version of the Universal Definition of Myocardial Infarction (UDMI). Other practical recommendations are given to overcome infrastructural barriers in the hospitals such as delayed laboratory reporting.

Therefore, this expert opinion article highlights strengths of the 2020 ESC NSTE-ACS Guidelines but also indicates limitations, where the recommendations are open to question in the light of inconsistent or absent evidence.

## Critical appraisal of the guidelines

### Strengths of 2020 ESC guidelines

Overall, the guideline authors must be congratulated for creating an extensive and comprehensive update of the preceding 2015 ESC Guidelines on acute coronary syndromes [2] without ST-segment elevations (NSTE-ACS). As a consequence of the accumulated evidence regarding the efficacy and safety of accelerated diagnostic protocols, new recommendations were introduced regarding the diagnostic strategies.

#### ESC 0/1 h protocol endorsed as the preferential diagnostic strategy

While the 2011 ESC [6] and 2015 ESC guidelines [2] endorsed the ESC 0/3 h protocol whenever hs-cTn assay were available in clinical routine, the 2020 ESC guidelines [1] now recommend to use the ESC 0/1 h protocol preferentially over the ESC 0/3 h protocol. Supportive evidence comes from several independent observational studies, a randomized controlled trial (RCT) from Australia [7], two real-world evidence studies [8, 9], and a meta-analysis from 15 trials that include 11,014 patients [10] that have conferred robust evidence on efficacy and safety of the ESC 0/1 h protocol. Besides the excellent discriminatory ability to rule out a NSTEMI, findings corroborate the safety of discharge after rule out of patients deemed to be at low risk [7–10]. One study shows higher discharge rates without increased utilization of coronary angiography, coronary interventions, cardiac stress, or imaging procedures [11]. The promotion of the ESC 0/1 h algorithm is further fostered by findings on reduced length of observation time and overall length of stay in ED, and lower hospital costs [12]. However, in the past, concerns were also expressed regarding the universal use of the ESC 0/1 h algorithm, mainly based on doubts regarding the accuracy of very small concentration changes, the limited evidence on early presenters, and uncertainties about the accuracy of rule-in using short re-testing intervals [13]. In full awareness that fast protocols are adopted slower than projected [14], the authors suggested solutions to overcome laboratory delays from blood draw to reporting, an issue presumed to represent the most important obstacle for worldwide implementation of fast diagnostic protocols. Therefore, the authors added a paragraph with a corresponding illustration proposing how to facilitate the diagnostic process by collection of blood at fixed intervals of 60 min without awaiting the report of the first blood draw. This strategy has the advantage of an optimal implementation of the ESC 0/1 h protocol. On the other hand, many perceive the additional costs and unnecessary blood collection anticipated in ~ 30% of low-risk patients who could have been ruled out by a single very low hs-cTn value < LoD at presentation as a relevant disadvantage of such a recommendation [15].

#### Deferred use of gender-, age- and other comorbidity-adjusted diagnostic cutoffs

There is an ongoing discussion on the importance of gender-, age-, or comorbidity-adapted decision cutoffs [16]. Beyond doubt and supported by findings from sophisticated cardiac imaging and cardiac function tests [17], hs-cTn concentrations were found to be lower in healthy women than in men resulting in a lower 99th percentile upper limit of normal (ULN) in women [18]. Likewise, concentrations increase with advancing age due to age-related subclinical comorbidities even in the absence of objective cardiovascular morbidity or renal impairment [19]. Since symptomatic patients presenting to an ED are usually older than the average age in healthy reference populations and are rarely healthy, there is a debate around the usefulness of a general uniform 99th percentile ULN. Concentrations of hs-cTn increase with age and comorbidities decreasing the numbers of patients with hs-cTn concentrations below the 99th percentile ULN on admission [20]. Hence, use of a low diagnostic threshold at the uniform 99th percentile is associated with a very high sensitivity but low clinical specificity and positive predictive value. The 4th Universal Definition of Myocardial Infarction (UDMI) recommended the use of sex-specific upper reference limits (URL, 99th percentiles) and the ESC endorsed the 4th UDMI [21]. Thus, the ESC 2020 Guidelines [1] should recommend using different 99th percentiles cutoffs for men and women. Surprisingly, this it is not the case. The authors justify this discrepancy between actual ESC documents with their clinical experience that a “mixture” of different ULN will confuse the clinicians’ judgement. Although, the 4th version of the UDMI [21] advocates the use of serial troponin measurements to discriminate chronic from acute myocardial injury, not all women having small hs-cTn increases, who are evaluated with a sex-independent ULN, may be correctly classified. While this issue may not be relevant for hs-cTnT [22] and some hs-cTnI assays, previous trials found a relevant diagnostic and prognostic re-classification in women with the Abbott Architect hs-cTnI assay [23]. With this assay, the difference between men and women is nearly two times larger than for hs-cTnT. Thus, the actual NSTE-ACS guidelines are in a partial conflict with 4th version of UDMI and might disadvantage women.

#### Increasing importance of high-sensitivity point-of-care troponin tests with designation for rule out

Until recently, point-of-care (POC) tests were recommended only in settings where central laboratory assays were not available, or when turn-around-times (TAT) exceeded 45–60 min. Due to an insufficient analytical sensitivity and precision of POC technologies, cTn testing on POC devices used to be utilized as an aid for rule-in of a NSTEMI, but its use for a reliable rule out of NSTEMI was discouraged [24]. The LSI Medience Pathfast hs-cTnI assay (formerly Mitsubishi Pathfast) meets high-sensitivity criteria [25], received approval by the FDA for use in clinical laboratory or POC settings [26], has been validated for the ESC 0/1 h protocol [27], and is also recommended in the 2020 ESC guidelines [1]. In perspective, several reports indicate a similar performance of other POC hs-cTnI tests and suggest that these assays could emerge as alternatives to centralized laboratory hs-cTn testing in the near future [27–30]. The shorter TAT with POC testing makes this technology more appealing as many patients can have AMI excluded at presentation or within 1 h.

However, several shortcomings should dampen the enthusiasm about POC testing including lack of evidence, despite the existing publications, that POC systems truly work in a real-world clinical practice when tests are run 24 h/7 days by non-laboratory personnel and using whole blood as material. In addition, the effect of analytical issues has not been addressed completely so far [29].

Finally, it is important to indicate that the list of available POC troponin assays is incomplete regarding cutoffs and concentration changes for several new hs-cTnI assays using the ESC 0/2 h protocol, and information on commercially available assays is not updated since it maintains the Singulex assay that it is not operational since 1 year.

#### New definition for high-sensitivity designation of cardiac Troponin assays

After the introduction of cTn assays with improved analytical sensitivity and precision, it became apparent that there were no agreed upon criteria to define the high-sensitivity designation. The scorecard criteria proposed by Fred Apple were reasonable as they combined analytical and clinical criteria [31]. Accordingly, cTn assays were attributed a hs-cTn designation if they were able to measure cTn at or below the 99th percentile value of a healthy reference population with a total imprecision of less than 10% CV, and were able to detect cTn in at least 50% of healthy individuals. This suggestion was refined by the International Federation of Clinical Chemistry (IFCC) and Laboratory Medicine Task Force on Clinical Applications of Bio-Markers (IFCC TF-CB) introducing the requirement to measure cTn concentrations above the limit of detection in 50% of men and women [32]. Unfortunately, reference populations with a sample size large enough to allow the calculation of sex-specific cutoffs are sparse, and many manufacturers have no access to appropriately sized sample banks. Now, the 2020 ESC guidelines [1] softened the IFCC criteria by eliminating the requirement for detection of cardiac troponin in at least 50% of both genders, presumably to facilitate the faster implementation of commercially available cTn assays with high-sensitivity designation [1]. Only in the POC paragraph a 50 to 95% rate of measurements above the LoD is briefly mentioned. However, at that point, a discussion should be initiated about standardized criteria for the validation of new hs-cTn assays and diagnostic algorithms before their entry in Guidelines.

### Controversies and uncertainties


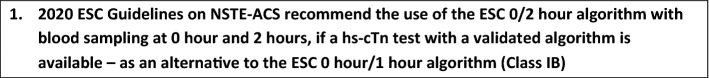


The 2020 ESC Guidelines recommend a novel ESC 0/2 h-algorithm as the preferred alternative to the ESC 0/1 h-algorithm in the early triage of suspected acute myocardial infarction This algorithm is similar to the ESC 0/1 h algorithm and uses distinct thresholds for baseline concentrations and change value for a re-testing at 2 h. The algorithm contains a strategy for immediate rule out based on a single low hs-cTn concentration at baseline and requires serial measurements two hours apart. While the two strategies combined in the novel algorithm have been derived and validated separately, the entire ESC 0/2 h algorithm has not been validated, yet. Distinct to the Accelerated Diagnostic Protocol (ADP) 0/2 h protocol, the ESC 0/2 h algorithm does not require a clinical score, i.e. the TIMI score to achieve an acceptable safety.

Five publications [33–37] were discussed to support this recommendation. Neumann et al. [33] prospectively evaluated individual patient-level data from 15 studies including 23,327 patients who presented to the emergency department with suspected acute myocardial infarction (AMI). The validation cohort on 13,047 patients included a 2-h hs-cTn-based ADP algorithm from Australia (summarized as ADAPT-BSN) and New Zealand (summarized as ADAPT-CH). Thus, while this study nicely supports the usefulness of fast diagnostic protocols with repeat sampling within 210 min, there is no obvious reason to restrict the recommendation to the ESC 0 h/2 h protocol and not to extend the recommendations to the hs-cTn-based 2-h ADP protocol, as well. Boeddinghaus et al. [34] compared the diagnostic accuracy, quantified by the area under the receiver operating curve (AUC), of the Siemens-hs-cTnI-Centaur assay versus the two established hs-cTn assays (Roche-hs-cTnT-Elecsys, Abbott-hs-cTnI-Architect). In addition, the investigators developed a diagnostic algorithm for the new Siemens Centaur assay for the ESC 0 h/1 h and ESC 0 h/2 h protocols. The derivation cohort for the hs-cTnI Siemens Centaur ESC 0 h/1 h algorithm was randomly selected among patients with an available blood sampling set at 0 h and 1 h. For the derivation set for the ESC 0 h/2 h algorithm patients were randomly selected in a 2:1 ratio to ensure a sufficient number of patients. Validation was executed in the same cohort but not in an independent external cohort. Optimal thresholds for rule out were selected to allow for maximal sensitivities and negative predictive values (NPVs) of 99% and were not based on package insert-specified thresholds. Optimal thresholds for rule-in were obtained based on a classification and regression tree (CART) analysis targeting a minimal positive predictive value (PPV) of 70%. While the performance of the ESC 0 h/1 h and ESC 0 h/2 h algorithm was studied for the new hs-cTnI Siemens Centaur in the derivation and validation set, with the Roche hs-cTnT and Abbott Architect hs-cTnI serving as reference, it was not within the scope of this study to compare the ESC 0 h/1 h and ESC 0 h/2 h algorithms. Thus, both algorithms have not been compared directly.

Reichlin et al. [35] analyzed the diagnostic accuracy of absolute delta (Δ) and relative (%) changes of cTn among 836 patients presenting to the emergency department with symptoms suggestive of AMI. Blood samples for the determination of high-sensitive cTnT and Siemens cTnI ultra were collected at presentation and after 1 and 2 h. The AUC for diagnosing AMI was significantly higher for 2-h absolute (Δ) versus 2-h relative (%) cTn changes. The authors concluded that absolute changes of cTn levels have a significantly higher diagnostic accuracy for AMI than relative changes and seem, therefore, to be the preferred criteria to distinguish AMI from other causes of cTn elevations.

Hence, neither the performance of hs-cTn in general nor the relative performance of the ESC 0 h/1 h versus the ESC 0 h/2 h was evaluated in this publication, raising the question why this article was referenced in the Guidelines to support a 0 h /2 h algorithm as an alternative to the ESC 0 h/1 h algorithm. Boeddinghaus et al. [36] developed an algorithm for the use of the Abbott Architect hs-cTnI assay in 1,435 patients using a derivation cohort from the Advantageous Predictors of Acute Coronary Syndrome Evaluation (APACE) study, and was consecutively validated for diagnostic accuracy in 1,194 patients from the 2-h Accelerated Diagnostic Protocol to Assess Patients With Chest Pain Symptoms Using Contemporary Troponins as the Only Biomarker (ADAPT) trial. Optimal thresholds for rule out were selected to allow for a maximal diagnostic sensitivity and NPV of 99%. Optimal thresholds for rule-in were selected to allow for the highest diagnostic specificity and positive predictive value (PPV). Diagnostic sensitivity and NPV were 98.7% and 99.7% for rule out, specificity and PPV were 97.4% and 82.2% for rule-in, respectively. Thirty-day survival was 100% for rule out patients in both cohorts. This study provides evidence supporting the safe use of a 0 h/2 h algorithm based on the Roche hs-cTnT and the Abbott Architect hs-cTnI assays. The algorithm was validated in an external independent cohort showing comparable performance. As such this article is valid to support the usefulness of 0 h/2 h algorithm, but does not provide information on its performance relative to the ESC 0 h/1 h or the ESC 0 h/3 h algorithms.

Nestelberger et al. [37] investigated an algorithm for the use of the ACCESS hs-cTnI (Beckman Coulter). The authors used 1,131 patients of a derivation cohort from the APACE study. The algorithm was consecutively validated for diagnostic accuracy externally in 1,280 patients from two studies using similar inclusion and exclusion criteria, namely the Accelerated Diagnostic Protocol to Assess Patients with Chest Pain Symptoms Using Contemporary Troponins as the Only Biomarker (ADAPT) and the Improved Assessment of Chest Pain Trial (IMPACT). Findings in the derivation and validation studies demonstrated safety and efficacy of the hs-cTnI-Access 0/2-h algorithm for rule out or rule-in of AMI. This study confers evidence for the usefulness of a 0/2 h algorithm but does not provide information on the relative performance of the 0/2 h algorithm compared to ESC 0 h/1 h or ESC 0 h/3 h algorithm.

Thus, consistent with 2020 ESC NSTE-ACS Guidelines [1], overall evidence supports a class IB recommendation for the ESC 0 h/2 h algorithm with the advantage that findings from observational studies were validated in external independent cohorts. Unfortunately, evidence supporting the effectiveness and safety of hs-cTn-based ADP protocols was not appropriately indicated leading to a disadvantage of the latter.

#### Underappreciation of the hs-cTn based ADP 0/2 h algorithms

Algorithms were developed based on hs-cTn results at admission and 2 h to further shorten evaluation time. These algorithms apply data-driven cutoffs not reflecting assay performance or biological plausibility, and incorporate specific (Δ) values. However, these alternative fast diagnostic strategies were not recommended as an alternative to the ESC 0 h/1 h or ESC 0 h/2 h protocols although there is abundant scientific evidence to support a class IB recommendation for ADP protocols, as well. Supportive evidence for the diagnostic performance of ADPs using hs-cTn with sampling at admission and 2 h is summarized in a review article by Eggers et al. [38].

The ADAPT trial investigated an ADP that was built on a TIMI risk score of 0, non-ischemic ECG, and non-increased cTn results at admission and at 2 h [39]. In studies using hs-cTn assays, the ADAPT-ADP provided a 100% sensitivity regarding 30-day MACE, but only 19.6–32.3% of the patients qualified for rule out. Modifying the ADP by including a TIMI score of 1 increased the rule out group to 29.8–41.5% at the expense of lower prognostic sensitivities (94.1–100%). Another critical point of this study is that no events occurred at all and, therefore, this study could have had significant selection bias or an error in the design.

The Emergency Department Assessment of Chest Pain Score (EDACS) integrates information on weighted variables (demographics, risk factors, symptom characteristics) [40]. The EDACS-ADP uses an EDACS score of 16 points, a non-ischemic ECG, and normal cTn concentrations at admission and at 2 h to identify patients eligible for early rule out. In a randomized head-to-head comparison with the ADAPT-ADP (*n* = 558), the EDACS-ADP pathway has been shown to identify more low-risk patients (47.7% vs 32.3%) while providing high safety with a sensitivity of 100% for survival. [41]. A randomized trial on 544 patients with suspected ACS randomized patients to a rapid diagnostic pathway or a standard care to test the effectiveness defined as discharge from hospital within 6 h without a major adverse cardiac event occurring within 30 days [42]. The impact of this randomized trial [42] on the strength of recommendation class and level of evidence was not appropriately addressed by the 2020 ESC Guidelines [1].

Wildi K et al. [43] directly compared the ADP 2-h protocol against the ESC 0/2 h protocol only for rule out (but not for rule-in) in two independent cohorts, namely the APACE study and the ADAPT trial. Both algorithms provided very high and comparable safety as quantified by the NPV and sensitivity for AMI and major adverse cardiac events (MACE) at 30 days in patients triaged toward rule out. The percentage of patients triaged toward rule out was significantly lower with the 2-h ADP (36–43%) versus the ESC 2-h algorithm (55–68%) with both assays and in both cohorts (*p* < 0.001). The sensitivity of the 2-h ADP was higher for 30-day major adverse cardiovascular events. The ESC 2-h algorithm was more efficient but not all patients ruled out for AMI by this algorithm were appropriate candidates for early discharge. Accordingly, the authors concluded that the 2-h ADP seems superior in the selection of patients for early discharge from the ED. Although this study cannot be regarded as appropriate for a recommendation of effectiveness across the entire diagnostic spectrum of suspected ACS, at least the favorable findings on the 2-h hs-cTn ADP algorithm raise the question why these study findings were excluded from the evidence-based recommendation process***.***

In summary, the substantial evidence supporting hs-cTn-based ADP protocols including the presence of positive findings from a randomized trial was not appropriately reflected by the 2020 ESC NSTE-ACS Guidelines [1]. Neither the existing evidence nor findings from a randomized trial [42] supporting a hs-cTnI based 2-h ADP was reported. In addition, the Guidelines did not mention unfavorable findings with ESC 0/2 h algorithm compared to the 2-h ADP from a study that directly compared strategies, with a restricted focus on rule out alone [43].

Thus, it appears that almost all recommendations and protocols have been derived from evidence based on the APACE registry while other alternative evidences were largely omitted.
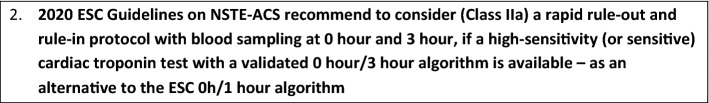


The 2015 ESC NSTE-ACS Guidelines [2] recommend use of the ESC 0 h/3 h algorithm based on several large observational studies that conferred evidence beyond doubt on the superiority of the ESC 0 h/3 h algorithm over the standard protocol with blood sampling at 0 h and 6–9 h in the absence of a high-sensitivity cardiac troponin assay. Four publications are now cited to support the assigned class IIa (LOE B) recommendation, i.e. to prioritize the ESC 0hour/1 h algorithm over the ESC 0 h/3 h protocol [44–47]. These references are likely to fuel a controversial debate as they do not unequivocally support the assigned class of recommendation. The article by Wildi et al. [44] evaluated the performance of the ESC 0 h/3 h protocol using four different high-sensitivity cTn assays. The 3-h rule-out protocol correctly diagnosed 99.9% (95% CI 99.1–100%), 99.5% (95% CI 98.3–99.9%), 100% (95% CI 98.1–100%), and 100% (95% CI 98.2–100%) of early presenters (< 6 h from chest pain onset) supporting a high recommendation class for the ESC 0 h/3 h over the “old” ESC standard protocol with blood sampling at 0 h and re-testing after 6–9 h. However, this article does not provide any data that compare the ESC 0 h/1 h or ESC 0 h/2 h protocols with the ESC 0 h/3 h protocol. Hence, citation of this article in this context seems inappropriate.

Badertscher et al. [45] directly compare the efficacy and safety of the ESC 0 h/3 h algorithm with the ESC 0 h/1 h algorithm for rule out of a MI using the Roche hs-cTnT and the Abbott Architect STAT hs-cTnI assays. The negative predictive values for the ESC 0 h/1 h algorithm are significantly lower than those for the ESC 0 h/3 h protocol using the Abbott Architect hs-cTnI. The NPVs for the ESC 0 h/3 h protocol are similar with the ESC 0 h/1 h algorithm when using the hs-cTnT assay. A significantly higher proportion of patients qualifying for rule out was demonstrated for the ESC 0 h/1 h algorithm versus the ESC 0 h/3 h protocol with both hs-cTnT and hs-cTnI. The relevance of this article for the ESC recommendation is controversial for two reasons: (a) the investigators focused on the rule-out part only, but did not evaluate the complete diagnostic process that incorporates rule-in AND rule out, as well as an observational zone that is exclusive to the ESC 0 h/1 h algorithm; (b) because the significant difference between ESC 0 h/1 h and ESC 0 h/3 h is restricted to a higher rate of patients in the rule-out pathway, and at least for the hs-cTnT assay a similar performance for the safety of rule out is demonstrated.

The third article by Chapman et al. [46] compared the diagnostic performance of three rapid diagnostic protocols, namely the High-STEACS pathway, the ESC 0 h/1 h, and the ESC 0 h/3 h protocols in a retrospective analysis. All three protocols were compared regarding sensitivities, specificities, negative and positive predictive values using the new Atellica IM hs-cTnI assay (Siemens Healthineer) from frozen samples. Briefly, the NPVs of all three strategies were between 98 and 99.5%, highest for the High-STEACS pathway and lowest for the ESC 0 h/3 h protocol. Sensitivities were considerably lower for all three strategies ranging from 90.8 to 92.2% for the ESC 0 h/3 h and ESC 0 h/1 h to 98% for the High-STEACS pathway. Specificities and PPVs were disappointingly low for all pathways, with the exception of a specificity of 98.2% for the ESC 0hour /1 h protocol. This comparative study was limited by three shortcomings: (a) it was retrospective evaluation; (b) the cutoffs of 3 and 5 ng/L for new Atellica IM hs-cTnI assay (Siemens) were transferred from a different study using hs-TnI concentrations measured by the Abbott Architect systems; (c) all measurements were conducted by Siemens Healthineers, and thus relevant conflicts of interest exist, which should preclude this study from being used in the Guidelines.

The fourth article by Chapman et al. [47] compared the performance of the High-STEACS pathway with the ESC 0 h/1 h protocol on 1,218 patients with suspected ACS. Briefly, this study is interesting but does not add any information on the superiority of the ESC 0 h/1 h protocol because this study compared the High-STEACS pathway, with blood sampling at 0 h and 3 h with the ESC 0 h/3 h protocol.
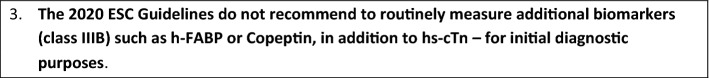


This class IIIB recommendation is most critical as it implies that other diagnostic biomarkers perform inferior or might even harm. Therefore, such a recommendation class should be supported by robust evidence, particularly when previous ESC guidelines [2] recommended using additional biomarkers, and because neither hs-cTn assays nor fast diagnostic protocols have been implemented broadly, at the moment [14].

#### Copeptin for instant rule out of MI

**Fig. 1:**
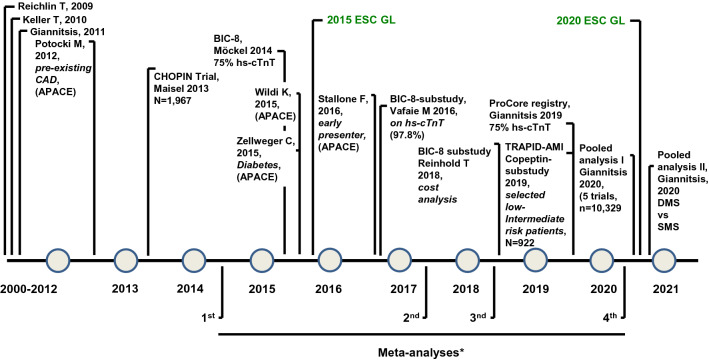
Accumulating evidence supporting the usefulness and added value of Copeptin in addition to cTn or hs-cTn over time

Copeptin is the molecule including the 39 aa carboxy-terminal (CT) sequence of the pro-vasopressin molecule and it is considered a non-specific biomarker with rapid increase in AMI in blood while cTn or hs-cTn levels are still normal, termed the “troponin-blind period” [54]. CT pro-vasopressin (Copeptin) as a marker of vasopressin reflects the immediate physiological response to arterial under-filling in AMI, when the cardiac output decreases in minutes after epicardial vessel closure. Commonly, this phenomenon is addressed as “cardiovascular stress”. The 2020 ESC NSTE-ACS guidelines [1] suggest that low Copeptin concentrations below the decision cutoff could improve the negative predictive value (NPV) of cTn for ruling out patients presenting early after symptom onset when cTn is not elevated in the first blood sample [1]. Conversely, a positive Copeptin while cTn or hs-cTn is below the 99th percentile URL suggests a strict serial troponin strategy. Despite increasing evidence [48–51] supporting that Copeptin accelerates the rule out of MI when combined with a hs-cTn assay (Fig. 1), ESC guidelines endorse the use of Copeptin as an alternative only when sensitive or high-sensitivity cardiac troponin assays are not available. This contrast with 2015 ESC Guidelines [2] that state that “Copeptin may have some added value even over high-sensitivity cardiac troponin in the early rule out of MI”. Recently, Wildi et al. [59] reported on the performance of 14 rule-out strategies in patients admitted with suspected NSTE-ACS. A dual marker strategy (DMS) combining Copeptin with hs-cTn was associated with the worst performance amongst all strategies for rule out in terms of sensitivities and NPVs, and was associated with the highest event rates within 90 days. However, it is important to indicate that DMS was tested retrospectively across the entire study cohort and did not exclude high-risk patients, as recommended [48, 49]. A substudy from TRAPID-AMI [55] investigating the role of Copeptin combined with hs-cTn elegantly demonstrated that exclusion of high-risk patients resulted in 100% sensitivity and 100%NPV, without any adverse outcome event. Accordingly, it does not become evident why the previously assigned recommendation was not expended but rather downgraded. Besides, the class III recommendation is contradicted as the Guidelines itself state that Copeptin can be used in special situations (page 13, right column).

#### Articles cited in support of an inferior performance of Copeptin in combination with hs-cTn

In the 2020 ESC NSTE-ACS Guidelines, six references [52–57] are listed to support a class IIIB recommendation. These references are summarized in Table 1***.***

**Table 1:** Literature claiming insufficient evidence for added value of Copeptin and h-FABP in addition to hs-cTn for the initial diagnosis (Adapted from Möckel ref#[82])

Authors	Marker	Results	Data source and patients
Boeddinghaus et al. 2017 [52]	Copeptin, hs-cTnT, hs-cTnI	PPV (off-label for copeptin) better with 1 h troponin	Retrospective from APACE, *n* = 1356
Hillinger et al. 2015 [53]	Copeptin, hs-cTnT at 0 and 1 h (off-label)	NPV 100% in copeptin and hs-TnT negatives	Retrospective from APACE 2006–2011, highly selected, * n* = 941, explorative study
Mueller et al. 2018 [54]	Copeptin		Opinion paper
Mueller-Hennessen et al. 2019 [55]	Copeptin, hs-cTnT	NPV 100% in low-risk cohort (label use)	TRAPID-AMI substudy, * n* = 922
Stallone et al. 2016 [56]	Copeptin, hs-cTnT	NPV in early presenters: Copeptin + hs-TnT 96% hs-TnT alone: 92.9%	Retrospective from APACE 2009–2011, *n* = 2183 and *n* = 328 from Luzern; *n* = 2000 analysed
O‘Donoghue et al. 2006 [57]	h-FABP	Independent prognostic value (death and MACE)	Retrospective from OPUS-TIMI-16; *n* = 2287

Most importantly, all referenced articles do not support an inferior diagnostic performance, and none of these articles indicate potential harm. In particular, the article by Boeddinghaus et al. [52] does not qualify for referencing because it focuses on the PPV of Copeptin which represents an off-label use of Copeptin in the setting of a suspected ACS. Among the four possible combinations, only the combination of normal or undetectable Copeptin and cTn/hs-cTn concentrations serve for rule out. In addition, the instant rule-out protocol should not include patients at high risk. In addition, previous studies were confounded by questionable use of statistical methods [53, 54, 58, 59]. First, comparison of non-independent groups, e.g. the same patients assessed for the performance of the 0-h versus 0-h/1-h protocol, should be tested with the McNemar instead of Pearson’s Chi^2^ test as the same population is tested repeatedly. Second, the evaluation of a rule-out test should be restricted to the assessment of sensitivities and NPVs but should not include specificities and PPV. Accordingly, C-statistics that assess the discriminatory ability of a continuous biomarker across the entire diagnostic spectrum, i.e. balance sensitivity and specificity are not appropriate.

The article by Hillinger [53] which derives data from the same APACE registry, demonstrates a NPV for Copeptin in combination with hs-cTnT of 100% and hence at least cannot support the claim of an inferior performance compared to the ESC 0 h/1 h algorithm.

The third article is a current opinion paper from the ESC Study Group on Biomarkers in Cardiology of the Acute Cardiovascular Care Association [54]**.** This educational paper does not opt against the measurement of Copeptin in addition to cTn or hs-cTn**.** Literally, it is stated that the added value of Copeptin to hs-cTn is less obvious, at the time of drafting that document. It is also stated that while studies found a marginal increase of overall diagnostic accuracy as quantified by the AUC, there was a statistically significant and clinically relevant increase of the NPV from 96 to 99%. The fourth article by Mueller-Hennessen et al. reports on the diagnostic and prognostic performance of Copeptin in addition to hs-cTnT in a substudy from the TRAPID-AMI trial [55]. Looking at the overall study cohort, a dual marker strategy (DMS) was associated with higher sensitivity (94.8 vs 89%) and negative predictive value (98.3 vs 97.4%) compared to the standard protocol based on the 99th percentile. After exclusion of high-risk patients as indicated by a modified HEART Score > 3 points, sensitivity and NPV of DMS increased to 100% for both, with no death occurring at 30 days. Hence, this TRAPID substudy rather supports the usefulness of DMS but definitely does not indicate potential harm. The fifth article by Stallone et al. [56] reports on findings from the APACE registry on 2,511 patients with suspected ACS presenting early after symptom onset. Of those, only 2000 patients were analyzed. In early presenters, sensitivities impressively increased from 74.5 to 91.2% and negative predictive values from 92.9 to 96% by the additional use of Copeptin on top of hs-cTnT. Thus, this article rather supports the usefulness of Copeptin in early presenters, presumably by overcoming the troponin-blind interval in the early hours after onset of myocardial infarction.

Finally, the article by Donohue et al. [57] reports findings from the randomized “Orbofiban in patients with unstable coronary syndromes-thrombolysis in myocardial infarction-16 (TIMI-OPUS-16) trial on 2,287 patients. This paper that was published 2006 and thus many years before the introduction of hs-cTn, demonstrated an independent prognostic value for heart-type fatty acid binding protein (h-FABP) for prediction of death and major cardiac events but does not confer any information on the diagnostic value of h-FABP or other additional biomarkers.

#### Articles in favor of a DMS combining Copeptin with high-sensitivity cardiac troponin

In addition to these controversies, numerous articles that confer incremental information on the added value of Copeptin to cTn and particularly to hs-cTn are not mentioned, at all (Fig. 1). These articles include the following investigations:

The Biomarkers in Cardiology-8 (BIC-8) trial [48], an international multicenter intervention trial on 902 patients that randomized patients with suspected ACS and low-to-intermediate risk to either the standard algorithm or the experimental DMS algorithm in the presence of a normal cTn/hsTn and a normal Copeptin [48]. This study demonstrated reduced length of stay in ED, higher discharge rates and importantly safety of discharge based on DMS that was as safe as discharge based on a standard diagnostic protocol. Of note, no death occurred in the experimental DMS arm at 30 days. This study was already presented during a Highlight Session at the 2014 ESC and corroborates the usefulness and safety of DMS based on a randomized trial design. In this RCT, Copeptin was combined with hs-cTn assays in about 2/3 of all patients. To generalize findings to clinical routine, a multicenter prospective observational trial (proCORE) was conducted in 18 emergency departments in nine European countries enrolling 2,451 patients with suspected ACS [49]. This registry confirmed the safety of the rule-out strategy with DMS with a significantly lower all-cause mortality than standard of care pathway with serial troponin measurements. Of note, there was only one fatality case in the DMS arm who died from cancer. Therefore, it is miraculous, why the RCT [48] confirmed by a multicenter registry was not considered for evidence at all. In addition, a health economic substudy [50] from the BIC-8 RCT demonstrated cost effectiveness using DMS versus standard diagnostic strategy in patients presenting with suspected ACS. Finally, a pooled analysis [51] using data on patient level was used aggregating data from 10,329 patients with suspected ACS who had received a rule out of MI using DMS or a standard troponin-based strategy. A sub-analysis of 3487 patients evaluating the hs-TropT from Roche showed a higher applicability with the DMS to rule-out patients when compared to a single marker strategy with hs-cTnT for instant rule out at admission. All four important publications [48–51] were not appropriately addressed in the 2020 ESC Guidelines. Not referring to important evidence yields an unbalanced recommendation.

#### Overemphasis on hs-cTn assays despite low global implementation of hs-cTn and fast protocols

The 2020 ESC guidelines continue to recommend the routine use of Copeptin as an additional biomarker for the early rule out of MI only in “the increasingly uncommon setting where hs-cTn assays are not available”. However, such a recommendation has no practical consequence in the light of the slow rate of global adoption of hs-cTn assays and fast protocols [14].

#### CK-MB for the diagnosis of a re-infarction, and myosin-binding protein C for the early rule out of NSTEMI

When hs-cTn assays are not available, the ESC guideline proposes as alternative biomarkers CK-MB for the diagnosis of a re-infarction, and Copeptin or myosin-binding protein C for the early rule out of NSTEMI. These proposals merit some comments.

##### CK-MB for re-infarction diagnosis

A re-infarction is defined as any acute myocardial infarction (AMI) occurring within 28 days of an incident or recurrent MI [60]. Thus, based on the release pattern of cTn and CK-MB, any MI occurring after 7–10 days of a previous MI will be detected more sensitively and specifically by any cTn method than by CK-MB.

The ESC Guidelines [1] refers to the use of CK-MB for early recognition of an MI occurring, supposedly, during the time interval in which cTn is still elevated owing to the first MI [1]. After any AMI, CK-MB values decrease to normal in 48–72 h, whereas cTn can remain elevated up to 7–10 days after the AMI. However, the guidelines do not take into account that CK-MB can be released by skeletal muscle in MI patients by several causes leading to loss of diagnostic specificity and that any it does not exist an unanimous value of the percent or absolute increase/fall that will define a significant CK-MB elevation after a previous MI. Of note, regardless the limited potential use of CK-MB as alternative for re-infarction the guidelines did not distinguish between CK-MB measured as catalytic activity or mass concentration. In addition, even when using contemporary methods for its measurement, cTn can detect re-infarctions occurring in the following 48-96 h after a previous MI using serial measurements [61].

Very recently, the ESC Study Group on Cardiac Biomarkers of the Association for Acute Cardiovascular Care published a current opinion article summarizing the reasons why CK-MB is no longer needed and suggests to eliminate CK-MB from the menu of biomarkers available for use in the evaluation of patients cardiovascular disease [62].

##### Cardiac myosin-binding protein C for earlier NSTEMI rule out

Cardiac myosin-binding protein C is a specific cardiac isoform (C-protein, MYBPC3, cMyBP-C, cMyC) which myocardial abundance is at least two times that of cTn. After an AMI, septal hypertrophy ablation or coronary artery bypass surgery, cMyC concentrations increase more rapidly and higher than those of cTn [63]. When measured with a so-called high-sensitivity immunoassay, its sensitivity and specificity for AMI diagnosis were comparable to that of hs-cTn [64], and the best cMyC diagnostic performance was observed in patients who presented very early after symptoms (< 3 h) [65]. Unfortunately, the methods available for cMyC measurement are only partly automatable and require several hours to the result and this fact precludes its use for the Guidelines proposed purpose.
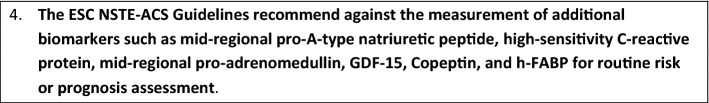


The recommendation against the routine use of additional biomarkers is based on three citations that exclusively refer to Copeptin but not to the other listed biomarkers [48, 66, 67].

The first citation refers to the Biomarkers-in-Cardiology-8 (BIC-8) trial [48], a randomized interventional trial that randomly assigned patients to either standard of care or to the experimental Copeptin arm where patients with negative troponin and Copeptin values at admission were eligible for discharge after final clinical assessment. Among the 902 low- to intermediate-risk patients, early discharge after clinical assessment in the Copeptin and Troponin negative arm was as safe as the standard diagnostic algorithm based on serial cTn or hs-cTn measurements with the 99th percentile as diagnostic threshold [48]. The testing of Copeptin is complementary to cTn and as such this study rather supports the additional measurement of Copeptin but definitely does not imply harm by a dual biomarker strategy. In a secondary analysis, Copeptin shows significant and independent prognostic values over hs-cTnT [66].

The second paper by Balmelli et al. [67] examined and compared the diagnostic and prognostic performance of selected cardiac biomarkers in 420 women and 827 men with suspected ACS recruited in the APACE study. Regarding the prognostic performance of selected biomarkers, the combination of cTnT and Copeptin outperformed cTnT alone, both in women and men. This study supports the additional use of Copeptin added to cTn but cannot be used to recommend against the use of Copeptin. The third paper based on the “Copeptin Helps in the early detection Of Patients with acute myocardial Infarction” (CHOPIN) trial [68] investigated the diagnostic performance of Copeptin added to conventional  cTn in suspected ACS presenting to an ED within 6 h of pain onset. A total of 1,967 patients with chest pain were enrolled at 16-sites study. The primary endpoint was diagnosis of AMI. The AUC of troponin alone in the first blood sample taken in the ED was 0.86, and increased to 0.97 by adding Copeptin. Using this double marker approach, a negative troponin and Copeptin < 14 pmol/l at presentation allowed AMI to be ruled out, with an NPV > 99% [68]. A second important result of the CHOPIN study relates to the prognostic role of Copeptin for outcome prediction at 30 days (*n* = 13 deaths; survival rate 99.3%), Copeptin was associated with adverse outcome, with a Chi-square test of 29.2 and a c-index of 0.872, and cTnI had a Chi-square value of 13.7 and a c-index of 0.828. Both markers were independent of each other and combining them provided significant added value (*p* = 0.01 for added value of cTnI, *p* < 0.0001 for added value of Copeptin). The incremental value was visible until the end of follow-up at 180 days. Hence, the findings from the CHOPIN trial [68] corroborate the clinical usefulness of a negative Copeptin in combination with a negative cTn but more importantly demonstrate added and independent prognostic value for prediction of outcomes within 180 days after NSTE-ACS. Additional evidence from observational trials [69–72] and a meta-analysis [73] have accumulated substantial evidence for a prognostic role of Copeptin when used together with a hs-cTn. Von Haehling [71] reported data from 2,700 patients with symptomatic coronary artery disease (CAD), who either presented with suspected ACS to the ED, or for elective coronary angiography. The predictive performance of Copeptin was independent of any other clinical variables or cardiovascular risk factors, and superior to that of troponin I or other cardiac biomarkers (*p* < 0.0001). Zellweger et al. [74] evaluated 379 patients with diabetes mellitus in a cohort of 1,991 patients presenting with suspected NSTE-ACS from the APACE registry. In multivariate Cox analysis, Copeptin, and hs-TnT were strong and independent predictors of 24-month mortality. Using the dual marker strategy (Copeptin and troponin) identified two groups of high-risk patients where 22.5% of the group with hs-cTnT and Copeptin above the cutoff died. The authors conclude that while Copeptin only slightly improves the early diagnosis of AMI provided by hs-cTnT, both markers (Copeptin and troponin) predict long-term mortality accurately and independently of each other. Potocki et al. [72] reported on 1,170 consecutive patients presenting with suspected AMI and pre-existing CAD. Copeptin used at a cutoff < 9 pmol/L was a strong and independent predictor of 1-year mortality, even after inclusion of hs-cTn into the Cox regression model with a HR 4.63 (1.83–11.71). Irrespective of hs-cTn or cTn levels, patients with low levels of Copeptin had an excellent prognosis compared with patients with raised levels of both Copeptin and cTn (360-day mortality 2.8–3.6% vs 23.1–33.8%, *p* < 0.001). Morawiec et al. [70] reported on 154 patients showing that the highest event-free survival at 30 days was achieved in patients stratified with an algorithm that combines hs-TnT, a modified HEART Score (mHS) ≤ 3, and Copeptin, with 100% (95% CI 75.3–100) NPV and 100% (95% CI 96.6–100) sensitivity. Another article by Reiter et al. [75] based on patients recruited in the APACE study reported on the diagnostic and prognostic value of biomarkers added to hs-cTn. In 1,074 patients evaluated for suspected NSTE-ACS, heart-type fatty acid binding protein (h-FABP) and Copeptin did not improve the diagnosis of patients but were found to add independent incremental prognostic information beyond hs-TnT. When adjusted to hs-cTnT levels, age, sex and cardiovascular risk factors, h-FABP had additional predictive value regarding mortality (HR 1.023 (95% CI 1.011 to 1.036), *p* < 0.001) beyond hs-cTnT (*p* > 0.05). This was also the case for Copeptin after adjustment (adj. HR 1.004 (95% CI 1.002 to 1.006), *p* < 0.001).
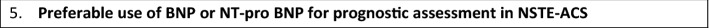


The 2020 ESC Guidelines [1] recommend that concentrations of BNP or NT-pro BNP should be used to gain prognostic information. The class IIa recommendation in favor of BNP or NT-pro BNP regarding prognostic information is based on three articles [76–78], a publication from the Study Group on Biomarkers in Cardiology [76] and two historic original publications dating back to 2001 [77] and 2003 [78], investigating the prognostic value of BNP or NT-pro BNP added to conventional cTn. More recent findings on the prognostic value of natriuretic peptides including BNP, NT-pro BNP, pro ANP or MR-pro ADM when added to an hs-cTn assay were not mentioned, at all. In the MERLIN-TIMI 36 trial [69] on 4,432 patients with NSTE-ACS who were randomized to treatment with ranolazine or placebo, MR-proADM and MR-proANP and Copeptin were found to add complementary prognostic information for CV death and HF in patients with NSTE-ACS performing as well as or better than BNP, cTnI, ST2, PAPP-A, and MPO (each *p* ≤ 0.01).
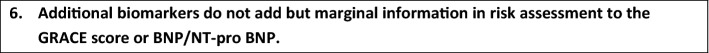


Recommendation of 2020 ESC Guidelines [1] on the marginal prognostic benefit of natriuretic peptides when added on top of the GRACE Score. The statement that additional biomarkers do not add significant but only marginal information for risk assessment to the GRACE risk score is not supported by existing evidence. Von Haehling [71] studied the role of Copeptin relative to the conventional GRACE Score (version 1) in a subgroup of 1,385 patients from a catheterization-laboratory cohort comprising 2,700 patients with symptomatic CAD. They reported a significant added value when Copeptin was added to the GRACE score compared to the GRACE score alone (AUC 0.718 vs 0.618, *p* < 0.00001). The AUC was higher than the model combining hs-cTnI Siemens ultra with the GRACE score (AUC 0.718 vs 0.623).

In an early investigation by Widera et al. in 1,122 patients with NSTE-ACS [79], that used a rigorous derivation/validation study design, GDF-15 was found to considerably add discriminatory information to the GRACE score (Version 1) with an increase in the AUC from 0.79 to 0.85 for the combined primary endpoint of death or non-fatal MI (the endpoint for which the score was developed). Adjustment of GRACE-predicted risks by GDF-15 led to a substantial proportion of patients appropriately being reclassified into higher or lower risks (a net 31% of the patients without events were reclassified into lower risk and a net 27% of patients with events were reclassified into higher risk), an effect size that can be classified as strong. In another study comparing the prognostic performance of 9 biomarkers and the GRACE score (Version 1) in 1,146 patients with NSTE-ACS [80], GDF-15 (AUC, 0.771), the GRACE score (AUC, 0.749), and NT-proBNP (AUC, 0.745) displayed the greatest discriminatory strength, and GDF-15 was the single biomarker that added most to the GRACE score. A recent study in 4,330 patients with NSTE-ACS enrolled in the MERLIN-TIMI 36 trial [81] using the new clinically available GDF-15 assay supports the conclusion that GDF-15 independently predicts risk in NSTE-ACS. It should be emphasized that the added value of biomarkers (including BNP/NT-pro BNP) to the new GRACE score (Version 2) has not been studied with the same methodological rigor. The conclusion that “additional biomarkers do not add but marginal information in risk assessment to the GRACE score or BNP/NT-pro BNP”, therefore, seems unjustified.
